# Mapping knowledge and comprehension of antimicrobial stewardship and biosecurity among veterinary students

**DOI:** 10.1371/journal.pone.0235866

**Published:** 2020-08-19

**Authors:** Zorana Kovacevic, Bojan Blagojevic, Jelena Suran, Olga Horvat

**Affiliations:** 1 Department of Veterinary Medicine, Faculty of Agriculture, University of Novi Sad, Novi Sad, Serbia; 2 Department of Pharmacology and Toxicology, Faculty of Veterinary Medicine, University of Zagreb, Zagreb, Croatia; 3 Department of Pharmacology Toxicology and Clinical Pharmacology, Faculty of Medicine, University of Novi Sad, Novi Sad, Serbia; University of Lincoln, UNITED KINGDOM

## Abstract

**Objectives:**

As an important public health concern, antimicrobial resistance (AMR) is related to lack of knowledge among healthcare professionals. Since the Global Action Plan on AMR highlights the importance of training all healthcare professionals, it is essential to focus our attention on the education related to judicious antimicrobial use. The current study was the first attempt in southeastern Europe to quantify the knowledge about antimicrobial usage and biosecurity measure among veterinary students.

**Methods:**

This questionnaire-based study was performed between April and May of 2019 on 213 veterinary students of the University of Novi Sad, Serbia and the University of Zagreb, Croatia.

**Results:**

Veterinary students appeared to be little aware of antimicrobial use in veterinary medicine contribution to overall AMR since only 56.8% have chosen strong contribution as the answer. Of the students surveyed, only 22.1%/35.7% of them strongly agreed/agreed that the amount of teaching time for pharmacology was about right. Students who denied having good knowledge of the pharmacology of antimicrobials showed higher knowledge about systemic use of antimicrobials in different clinical scenarios (p = 0.002). High importance of some antimicrobials for human medicine was not recognized by surveyed students. Only 8.5% of them identified gentamicin correctly, as first-line therapy. Students expected to graduate later were more likely to identify the importance of rating antimicrobials correctly than those who thought they would graduate earlier (p = 0.002). More than half of students gave correct answer at scenario regarding a dog with recurrent pyoderma by choosing culture and susceptibility (C & S) testing. Our students who think they will graduate sooner have higher knowledge level on C & S testing sample submission for range of clinical scenarios (p = 0.004). Moreover, appropriate use of PPE (personal protective equipment) procedure and biosecurity measure were reported for two thirds of our students in case of only for two clinical scenarios.

**Conclusion:**

This study reveals that among veterinary students from Croatia and Serbia improved undergraduate education is needed on the AMR with emphasis on antimicrobial stewardship (AMS) and appropriate biosecurity.

## Introduction

Antimicrobial Resistance (AMR) is recognized as a serious public health challenge in both human and veterinary medicine [1–3]. Prudent antimicrobial use (AMU) is therefore essential to preserve the effectiveness of these important drugs [4] as usage which maximizes therapeutic efficacy of antimicrobials and minimizes the development of AMR [5–7].

The term antimicrobial stewardship (AMS) is relatively new and is defined as a coherent set of actions promoting the use of antimicrobials responsibly [8] and it involves strategies to ensure that they are used effectively [9,10]. The necessity to guide AMU via stewardship was first recognized in human medicine [11]. In Serbia and Croatia, various efforts in rational AMU implementation and in hospital-based AMS programs development in human medicine have been exerted [12,13].

According to total antibiotic consumption in 2015 (expressed in number of defined daily dose per 1000 inhabitants per day), Serbia (31.57) and Croatia (20.28) are among European countries with the highest antibiotic consumption rate, although data about this consumption in veterinary practice are insufficient and the follow-up of this consumption itself are at an early stage in these two countries [14]. Advancement and implementation of AMS programs is important in both human and veterinary medicine. Hence, tackling AMS in veterinary practice and prudent AMU represents one of major possibilities to decrease AMR [15–17]. Since human, animal and environmental resistance genes are interconnected [18–20] and united in the One health approach [21–23] it is important to train all healthcare providers on AMR issue. In addition, AMS interventions may be more successful if they are introduced as early as at the undergraduate level [24]. All healthcare students can play crucial role in reducing AMR, which is established in the WHO Action plan on AMR [25]. Moreover, in the WHO principles [5] is pointed out that veterinary undergraduate, postgraduate and continuing education should be evaluated to ensure that preventive medicine, prudent AMU and AMR are given high priority. In addition, implementing biosecurity measures in veterinary practice in a suitable way could have influence on AMU decrease [26,27]. Moreover, since biosecurity refers to all hygienic practices designed to prevent occurrences of infectious diseases [28], having insight in veterinary students' knowledge on biosecurity behavior could play important role in AMR reduction. Even substantial scientific progress has been made on the objective assessment of farm biosecurity to reduce antimicrobial usage in food animal production [29], more education in ensuring that biosecurity practices are implemented is needed [30,31]. Recently published data addressing AMR have been focused on medical students [32–35]. A survey conducted in Serbia and Croatia was about teaching medical students how to prescribe antibiotics prudently [36]. Moreover, published studies examined pharmacy and medical students in Croatia [37] and the general population in Serbia [38] on AMR issue, but none was focused on veterinary students in southeastern Europe.

Therefore, the aim of this study was to assess knowledge and comprehension regarding AMS and biosecurity measures among veterinary students in Serbia and Croatia as prospective prescribers.

## Materials and methods

### Setting

The study was conducted at the University of Zagreb, being the largest in Croatia and at the University of Novi Sad, the second largest one in Serbia, between April 1^st^ and May 30^th^ of 2019. Faculty of Veterinary medicine in Zagreb is the only one in Croatia while in Serbia there are two of them, in Belgrade and in Novi Sad. For that reason, these findings might not be generalizable for veterinary students all over Serbia. However, based on the fact that a curriculum of these two faculties in Serbia is uniform, our sample can be considered representative for these two countries. Furthermore, larger-scale study is needed where veterinary students from other neighboring countries would define further insights into how culture of antibiotics and biosecurity is working in this part of Europe. The target population included fourth, fifth and sixth year of students from both universities, Faculty of Veterinary Medicine in Zagreb (n = 135) and Faculty of Agriculture, Department of Veterinary Medicine in Novi Sad (n = 78) after they had received and completed teaching on antibiotics in their pharmacology course at the third year of studies. The curriculum of these faculties takes six years and contents related to AMU and AMR are mainly incorporated in chapters of pharmacology and microbiology courses. The sample size, which was the total number of registered veterinary students of selected years was 329; veterinary students from Croatia (n = 242) and from Serbia (n = 87). All students from the both universities, were eligible to participate in the study. The study was conducted with the approval of the dean of the both faculties. The questionnaire was distributed to all students attending randomly selected lectures at these faculties. Besides, the permission of the concerned lectures was also taken. The study sample students were approached and asked to complete the questionnaire during the last 15 minutes of their scheduled class. Before the administration of questionnaires, the background and intentions of the survey were thoroughly explained. After giving their written informed consent, students were instructed by the researchers on how to complete the questionnaire without any undue pressure, with high level of confidentiality and anonymity throughout the study.

The study was approved by the Ethical Committee of the Faculty of Agriculture in Novi Sad (approval number 9000/1160/2) before the research was conducted.

### The questionnaire

The questionnaire (S1 Questionnaire) used in this study is based on the questionnaire of Hardefeldt et al. [39]. Necessary modifications regarding the level of knowledge about the guidelines on AMR, biosecurity and veterinary drugs were made to enable correct answers to these questions since there are differences at national level among countries. The questionnaire was originally drafted in English, then translated into Serbian and Croatian, and pilot tested for consistency, readability, design and comprehension by 15 veterinary students from each University. The questionnaire was divided in six sections (available as Supplementary Materials). The first section consisted of questions referring to the demographic and academic characteristics about the respondents such as age, gender, year of study, year of graduating and area of interest. Additionally, the first section asked their opinion on the degree to which veterinary antimicrobial use contributes to overall AMR. The second section consisted of 16 antimicrobials where respondents were asked to indicate whether the named antimicrobials were first, second or third line therapies (as determined by the World Health Organization—Critically important antimicrobials for human medicine) [40] whereby critical importance rating agents classified as first line therapies, highly importance rating agents classified as second line, and important rating agents classified as third-line therapies. The third section consisted of 17 specific clinical scenarios where students were asked to choose the frequency (always, often, sometimes, rarely, never, or not sure) with which they would use antimicrobials. The fourth section included 17 specific scenarios where respondents were asked to indicate the frequency (always, frequently, rarely, never, or not sure) with which they would submit samples for culture and susceptibility (C & S) testing. Depending on the scenario, answers either always or never were correct. The fifth section consisted of 19 different scenarios where students had to indicate the level of biosecurity they would apply for each scenario. Among to the proposed answers, according to the question, correct answers were gloves & gown/overalls, or gloves, gown/overalls & face protection or gloves, overalls with head protection, P2 respiratory mask and goggles. Opinion on the quantity and quality of teaching on AMS within their curricula and knowledge about different guidelines was the subject of the sixth section. There was a difference in these questions in the questionnaires distributed in Serbia and Croatia since the guidelines on AMR and biosecurity, as well as the Law on Medicines and Medicinal Devices were changed in accordance with the guidelines and law adopted in both countries at the national level, but they referred to the same content.

### Data analysis

Descriptive and comparative statistical data analysis was performed with the IBM SPSS Statistics 22 (SPSS Inc., Chicago, IL, USA). Descriptive statistical methods and methods for modeling the relationship between outcomes and potential predictors were used to analyze the primary data. As for the descriptive statistical methods, the following were used: measures of central tendency (arithmetic mean), measures of variability (standard deviation), and relative numbers (indicators of structure) as absolute numbers (n) and percentage representation. The relationship of dependent variables (knowledge scores in individual questions) with potential predictors was modeled by linear regression adjusted for knowledge scores on another group of questions. Multivariate logistic regression included those predictors from univariate analysis statistically significant at the significance level of 0.05. Statistical hypotheses were tested at a level of statistical significance (alpha level) of 0.05.

## Results

### Sociodemographic and academic characteristics of respondents

Out of 329 questionnaire distributed, the survey was completed by 213 students (response rate 64.74%). The average age of students at both University of Novi Sad and University of Zagreb was 23.88±1.64 years. About two-thirds (67.6%) of the total 213 students were females. The majority of students were in the fourth (41.8%) and fifth (48.8%) year of their academic study. Only 9.4% of them were in their sixth year of study and 39.4% of the respondents thought they would finish their studies in 2021. Almost one-third of the students (30.1%) did not decide yet about their area of interest, while most of them said they would choose small animals practice (43.2%). Additionally, similar number of them choose farm animals practice (13.2%) and the remaining would prefer working in public health, government, industry or research area (in total 12.7%). All sociodemographic and academic characteristics of the respondents are outlined in Table 1.

**Table 1:** Sociodemographic and academic characteristics of veterinary students at University of Novi Sad (VSNS) and University of Zagreb (VSZG).

Characteristics	Total sample
	n (%)
**Age (years)**	
22	5 (2.3)
23	75 (35.2)
24	99 (46.5)
25	14 (6.6)
26	13 (6.1)
27	3 (1.4)
29	4 (1.9)
31	1 (0.47)
32	1 (0.47)
34	1 (0.47)
**Gender**	
male	69 (32.4)
female	144 (67.6)
**Year of study**	
4	89 (41.8)
5	104 (48.8)
6	20 (9.4)
**Year of graduating**	
2020	65 (30.5)
2021	84 (39.4)
2022	64 (30)
**Area of interest**	
Small animal	92 (43.2)
Farm animal	28 (13.2)
Public health, government, industry, research	27 (12.7)
Undecided	31 (14.6)

### Knowledge on AMR and guidelines

All students were asked if veterinary use of antimicrobials contributed to overall AMR and if so, to which extent. More than half of the respondents (56.8%) choose strong contribution. The rest of respondents showed poor knowledge on antibiotic use by choosing moderate (36.6%), minimal (12.7%) and no contribution (30.1%) to overall AMR (Table 2). About 56.8% of students heard about National Strategy for the control of bacterial resistance to antibiotics in both countries and referred to it often. Around two thirds of respondents were familiar with the Law on Medicines and Medicinal Devices and often referred to it. In addition, as many as 70% of students from both countries heard about biosecurity guideline (Table 2).

**Table 2:** Knowledge on antibiotic use and level of knowledge about the different guidelines.

Questions	Total sample
	n (%)
**Veterinary use of antimicrobials contributions to overall AMR**	
strong	121 (56.8)
moderate	78 (36.6)
minimal	1 (12.7)
no contribution	4 (1.9)
not sure	9 (4.2)
**National Antimicrobial Resistance Strategy in Serbia and Croatia**	
know of, refer to often	121 (56.8)
have read, never heard of	92 (43.2)
**The low on medicines and medicinal devices**	
know of, refer to often	158 (7.2)
have read, never heard of	55 (25.8)
**Policy on animal health measures against infections and parasitic diseases and their financing in 2019**	
know of, refer to often	149 (70.0)
have read, never heard of	64 (30.0)

### Knowledge on antibiotic use in animals

**Fig 1:**
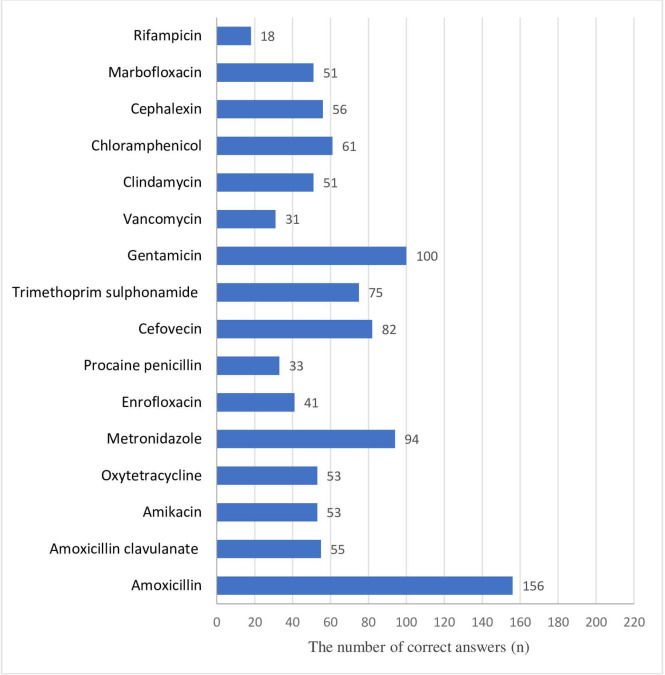
The number of correct answers given by veterinary students (n = 213) correctly identifying the level of importance of antimicrobials in human medicine (first, second or third line therapy).

Students were asked to decide if antimicrobials given in Fig 1 should be first, second or third line therapy. Most of the students gave correct answers identifying amoxicillin as first-line therapy (73.9%), while the smallest number of them recognized rifampicin as first-line therapy (8.5%) (Fig 1). Besides, less than half of respondents correctly identified gentamicin (46.9%) as first-line therapy, while chloramphenicol (second line) was incorrectly categorized by 71.4% of veterinary students (Fig 1).

**Fig 2:**
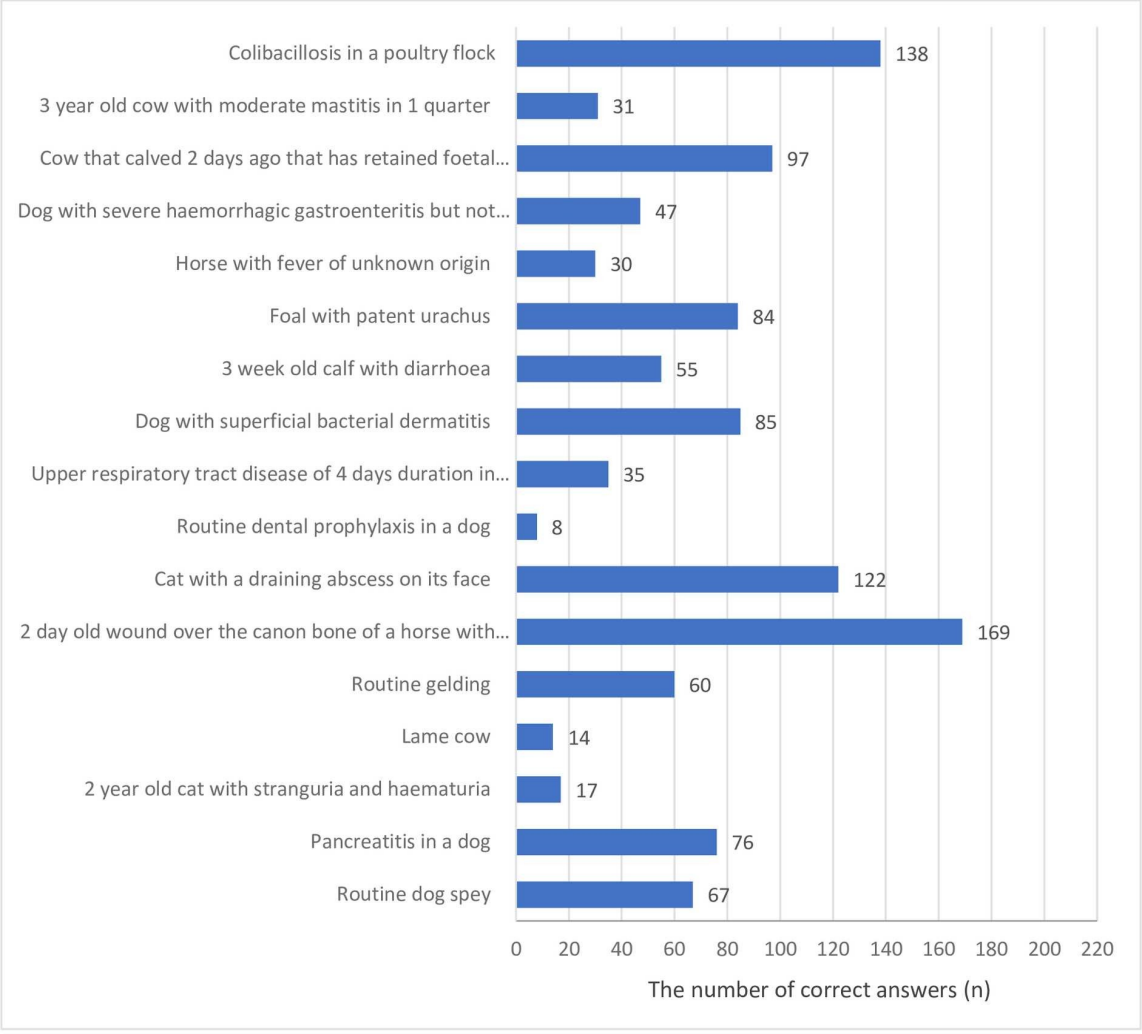
The number of correct answers given by veterinary students (n = 213) responding to a survey would treat a range of clinical scenarios with systemic antimicrobials (injectable or oral).

The students were asked to indicate how frequently they would use (always, frequently, rarely, or never, or not sure) systemic antimicrobials for a range of clinical scenarios. All scenarios were designed in such a way that systemic antimicrobials were rarely or never indicated. The vast majority of respondents (79.3%) gave the correct answers (rarely or never) regarding a 2-day old wound over the canon bone of a horse with the bone exposed, whereas the routine dental prophylaxis in a dog was the scenario with the smallest number of correct answers (rarely or never) among veterinary students (3.8%) (Fig 2).

### Rational antimicrobial use and biosecurity

**Fig 3:**
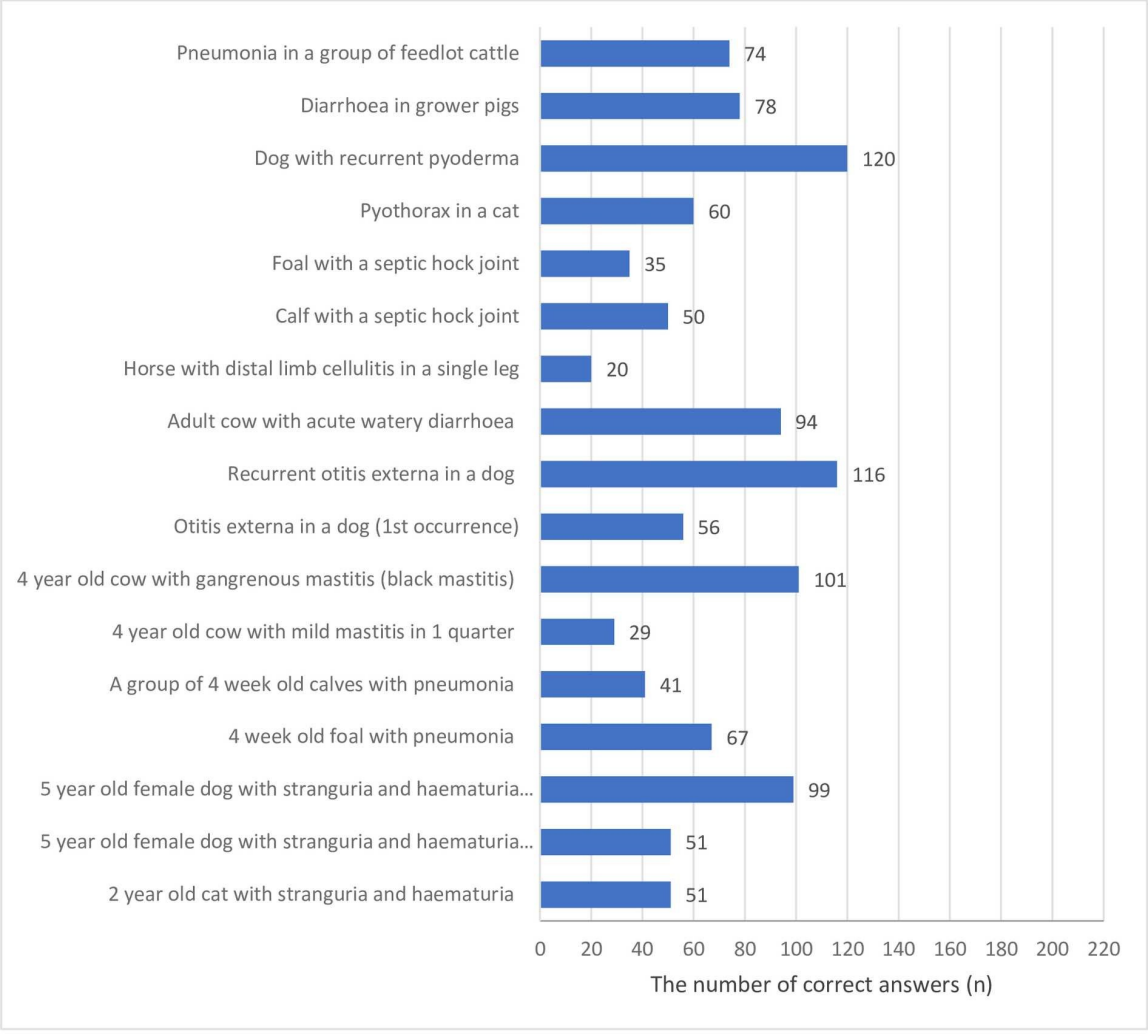
The number of correct answers given by veterinary students (n = 213) responding to a survey would perform culture and susceptibility for a range of clinical scenarios.

In order to access rational antimicrobial use, students were asked how frequently (always, frequently, rarely, or never) they would perform C & S testing for a range of clinical scenarios. The majority of them gave correct answer (always or frequently) regarding C & S testing in a dog with recurrent pyoderma (56.3%), while a horse with distal limb cellulitis in a single leg was the scenario with the smallest number of correct answer (9.4%) (Fig 3).

**Fig 4:**
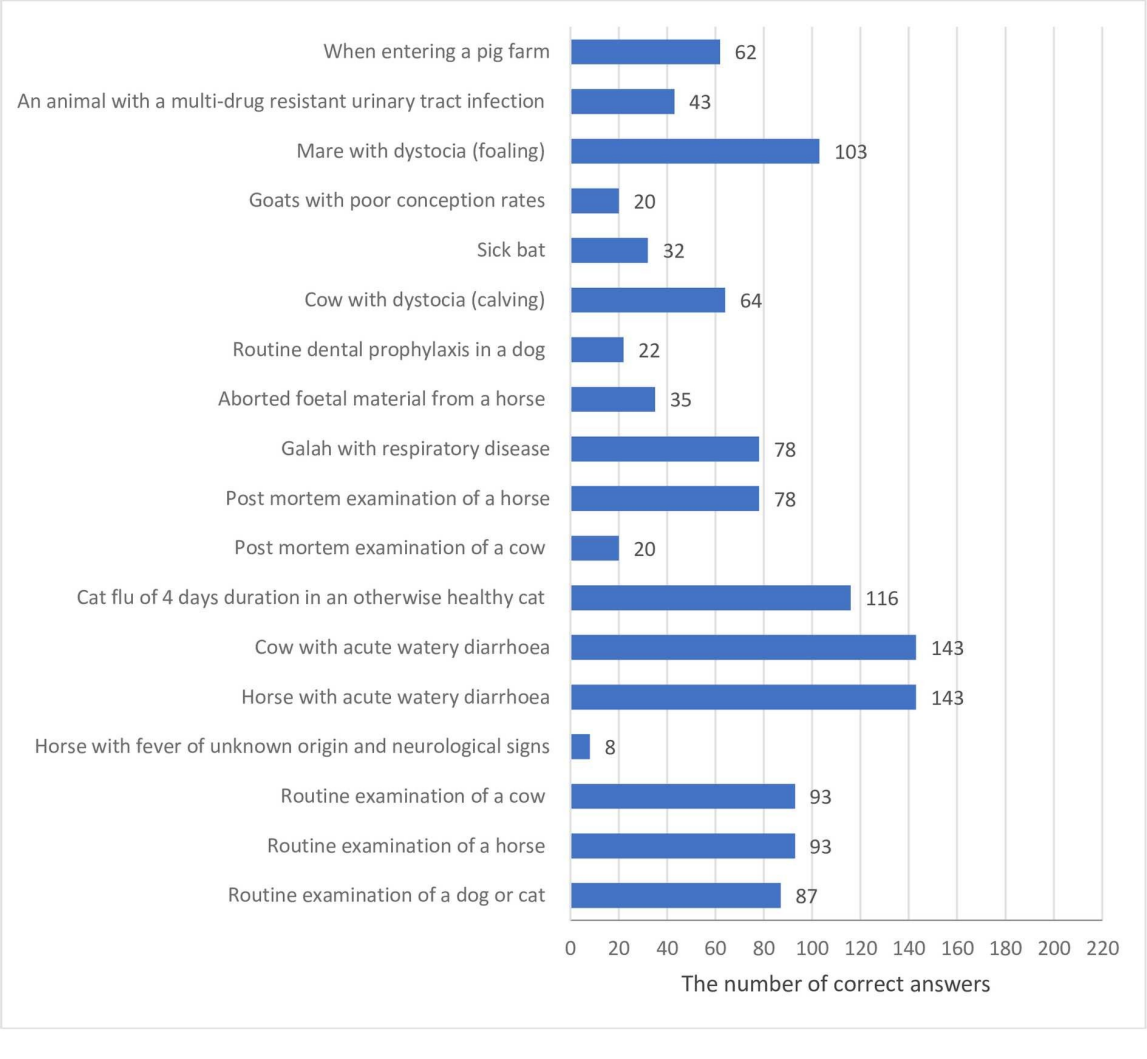
The number of correct answers given by veterinary students (n = 213) responding to a survey applied appropriate biosecurity for a range of clinical scenarios.

Veterinary students were asked to choose appropriate use of biosecurity and PPE (personal protective equipment) procedure for a range of clinical scenarios. Two thirds of students gave correct answer for the scenario related to a cow with acute watery diarrhoea (67.1%), while the lowest percentage of respondents reported appropriate use of PPE and biosecurity on the scenario regarding horse with fever of unknown origin and neurological signs (3.8%) (Fig 4).

### Quantity and quality of teaching on AMS among veterinary students

Of the students surveyed, only 14.1% and 34.7% of respondents strongly agreed and agreed, respectively that prudent antimicrobial use was taught adequately; 22.1% of them strongly agreed, while 35.7% agreed that the amount of teaching time for pharmacology was about right. Contrary to this findings, 10.3% and 42.3% of students strongly agreed and agreed, respectively that they had good knowledge on the pharmacology of antibiotics. In the follow-up questioning, most of them strongly agreed (40.8%) and agreed (46%) on having good understanding of the mechanisms of AMR. As for understanding of AMS, 67.6% and 28.2% of students strongly agreed and agreed, respectively to this statement. Moreover, 16.4% of respondents strongly agreed and 46.9% of them agreed that they knew how to use antibiotics to minimise the risk of AMR developing (Table 3).

**Table 3:** Veterinary student's opinions responding to a survey (n = 213) about the teaching of antimicrobial resistance and antimicrobial stewardship.

	Strongly agree n (%)	Agree n (%)	Neither agree nor disagree n (%)	Disagree n (%)	Strongly disagree n (%)
a. I understand what AMS is	144 (67.6)	60 (28.2)	4 (1.9)	4 (1.9)	1 (0.5)
b. The amount of teaching time for prudent AMU is about right	30 (14.1)	74 (34.7)	14 (6.6)	83 (39)	12 (5.6)
c. I understand AMR mechanisms	87 (40.8)	98 (46)	8 (3.8)	20 (9.4)	0
d. I have a good knowledge of the pharmacology of antibiotics	22 (10.3)	90 (42.3)	25 (11.7)	67 (31.5)	9 (4.2)
e. The amount of teaching time for pharmacology is about right	47 (22.1)	76 (35.7)	32 (15)	48 (22.5)	10 (4.7)
f. I know how to use antibiotics to minimise the risk of AMR developing	35 (16.4)	100 (46.9)	28 (13.1)	42 (19.7)	8 (3.9)

*AMR, antimicrobial resistance; AMS, antimicrobial stewardship; AMU, antimicrobial use

Multivariate linear regression model included all predictors of knowledge score within the 5th group of questions that were statistically significant (p = 0.05) in univariate models. The model contained 2 predictors, as shown in Table 4.

**Table 4:** Univariate and multivariate linear regression with total knowledge score within the 5th group of questions (For each of the following antimicrobials, please indicate if they are for first, second or third line therapy) as a substitute variable adjusted for knowledge scores on other group of questions.

Variable	Univariante		Multivariante	
	B	p	B	p
Gender	-0.612	0.049	-0.500	0.110
Age	-0.180	0.081		
Grade year	-0.239	0.293		
Year of graduating	0.457	0.015	0.407	0.032
Area of interest				
Undecided	Reference category			
Small animal	-0.024	0.945		
Farm animal	0.085	0.858		
Public health, government, industry, research	0.441	0.360		
Veterinary use of antimicrobials contributions to overall AMR	-0.037	0.877		
Knowledge on National Strategy for the control of bacterial resistance to antibiotics	-0.063	0.833		
Knowledge on the low on medicines and medicinal devices	-0.380	0.248		
Knowledge on the policy on animal health measures against infections and parasitics diseases and their financing in 2019	-0.464	0.140		
a. I understand what antimicrobial stewardship is	-0.060	0.782		
b. The amount of teaching time for prudent antibiotic use is about right	-0.056	0.634		
c. I understand AMR mechanisms	-0.056	0.730		
d. I have a good knowledge of the pharmacology of antibiotics	-0.084	0.524		
e. The amount of teaching time for pharmacology is about right	-0.193	0.122		
f. I know how to use antibiotics to minimise the risk of AMR developing	-0.128	0.341		

The model as a whole was statistically significant (p = 0.002). There was no multicollinearity between predictors. In the multivariate linear regression model, the question “What year do you think you will graduate if everything goes according to the plan?” was a statistically significant predictor of higher knowledge score in the 5th group of questions, adjusted for knowledge score in other question groups (B = 0.407; p = 0.032). The students who thought they would graduate later were more likely to correctly identify the rating importance of antimicrobials.

Multivariate linear regression model included all predictors of knowledge score within the 6th group of questions that were statistically significant (p = 0.05) in univariate models. The model contained 3 predictors, as shown in Table 5. The model as a whole was statistically significant (p < 0.001). There was no multicollinearity among predictors. In the multivariate linear regression model, the variables “National Strategy for the control of bacterial resistance to antibiotics” (B = 0.613; p = 0.034) and “I have good knowledge of the pharmacology of antibiotics” (B = - 0.320; p = 0.011) were statistically significant predictors of higher knowledge score in the 6th group of questions, adjusted for knowledge score in other question groups. The students who frequently referred to the National Strategy for the control of bacterial resistance to antibiotics and who did not agree that they had a good knowledge of the pharmacology of antimicrobials were more likely to propose appropriate prescribing of antimicrobials in different scenarios.

**Table 5:** Univariate and multivariate linear regression with total knowledge score within 6th group of questions (For each of the following scenarios please indicate if you think systemic (injectable or oral) antimicrobials are indicated) as a substitute variable adjusted for faculty and knowledge scores on other group of questions.

Variable	Univariante		Multivariante	
	B	p	B	p
Gender	-0.126	0.688		
Age	0.205	0.044	0.145	0.148
Grade year	0.090	0.689		
Year of graduating	-0.092	0.627		
Area of interest				
Undecided	Reference category			
Small animal	-0.122	0.719		
Farm animal	0.364	0.438		
Public health, government, industry, research	-0.607	0.202		
Veterinary use of antimicrobials contributions to overall AMR	0.366	0.130		
Knowledge on National Strategy for the control of bacterial resistance to antibiotics	0.796	0.006	0.613	0.034
Knowledge on the low on medicines and medicinal devices	0.034	0.917		
Knowledge on the policy on animal health measures against infections and parasitics diseases and their financing in 2019	0.011	0.971		
a. I understand what antimicrobial stewardship is	-0.322	0.132		
b. The amount of teaching time for prudent antibiotic use is about right	-0.104	0.370		
c. I understand AMR mechanisms	-0.075	0.641		
d. I have a good knowledge of the pharmacology of antibiotics	-0.398	0.002	-0.320	0.011
e. The amount of teaching time for pharmacology is about right	-0.216	0.080		
f. I know how to use antibiotics to minimise the risk of AMR developing	-0.251	0.057		

Multivariate linear regression model included all predictors of knowledge score in the 7th group of questions that were statistically significant (p = 0.05) in univariate models. The model contained 3 predictors, as shown in Table 6. The model as a whole was statistically significant (p = 0.004). There was no multicolinearity among predictors. In the multivariate linear regression model, the following variables Gender (B = 0.669; p = 0.046) and If all goes well, in which year will you graduate? (B = - 0.507; p = 0.008) were statistically significant predictors of higher knowledge score in the 7th group of questions, adjusted for knowledge score in other questions groups. Female students and those who thought they would graduate sooner were more likely to perform always or frequently C & S testing in 17 different scenarios.

**Table 6:** Univariate and multivariate linear regression with total knowledge score within 7th group of questions (For each of the following scenarios please indicate if you would submit samples for culture and susceptibility testing) as a substitute variable adjusted for faculty and knowledge scores on other group of questions.

Variable	Univariante		Multivariante	
	B	p	B	p
Gender	0.896	0.004	0.669	0.046
Age	0.184	0.081		
Grade year	0.418	0.070		
Year of graduating	-0.599	0.002	-0.507	0.008
Area of interest				
Undecided	Reference category			
Small animal	0.751	0.030	0.535	0.119
Farm animal	0.292	0.545	0.471	0.321
Public health, government, industry, research	0.240	0.624	0.295	0.540
Veterinary use of antimicrobials contributions to overall AMR	-0.080	0.746		
Knowledge on National Strategy for the control of bacterial resistance to antibiotics	-0.052	0.866		
Knowledge on the low on medicines and medicinal devices	0.247	0.464		
Knowledge on the policy on animal health measures against infections and parasitics diseases and their financing in 2019	0.483	0.134		
a. I understand what antimicrobial stewardship is	-0.098	0.661		
b. The amount of teaching time for prudent antibiotic use is about right	-0.047	0.698		
c. I understand AMR mechanisms	-0.137	0.411		
d. I have a good knowledge of the pharmacology of antibiotics	0.085	0.525		
e. The amount of teaching time for pharmacology is about right	0.113	0.379		
f. I know how to use antibiotics to minimise the risk of AMR developing	-0.108	0.433		

In univariate linear regression models, the variable National Strategy for the control of bacterial resistance to antibiotics (B = 1.303; p = 0.001) was the only statistically significant predictor of higher knowledge score in the 8th question group, adjusted for knowledge score in other question groups. The students who often referred to National Strategy were more frequently used PPE procedure and biosecurity (Table 7).

**Table 7:** Univariate and multivariate linear regression with total knowledge score within 8th group of question (For each of the following scenarios please indicate the level of biosecurity you would take for examination and performing procedures) as a substitute variable adjusted for faculty and knowledge scores on other group of questions.

Variable	Univariante		Multivariante	
	B	p	B	p
Gender	0.092	0.836		
Age	0.088	0.544		
Grade year	-0.056	0.860		
Year of graduating	0.184	0.491		
Area of interest				
Undecided	Reference category			
Small animal	-0.021	0.965		
Farm animal	-0.285	0.670		
Public health, government, industry, research	0.190	0.779		
Veterinary use of antimicrobials contributions to overall AMR	0.343	0.305		
Knowledge on National Strategy for the control of bacterial resistance to antibiotics	1.303	0.001		
Knowledge on the low on medicines and medicinal devices	0.207	0.654		
Knowledge on the policy on animal health measures against infections and parasitics diseases and their financing in 2019	0.255	0.565		
a. I understand what antimicrobial stewardship is	-0.351	0.246		
b. The amount of teaching time for prudent antibiotic use is about right	0.117	0.475		
c. I understand AMR mechanisms	-0.176	0.438		
d. I have a good knowledge of the pharmacology of antibiotics	-0.045	0.805		
e. The amount of teaching time for pharmacology is about right	0.097	0.579		
f. I know how to use antibiotics to minimise the risk of AMR developing	-0.087	0.646		

## Discussion

To the best of our knowledge, there are no published data among veterinary students in Serbia and Croatia about knowledge that guide AMS and biosecurity among them as an important part in counteracting the problem of AMR.

As in humans, use of antimicrobials in veterinary practice contributes to the rise and spread of AMR all over the world [19, 41, 42]. Besides AMU in food animals, pet animals could transfer resistant bacteria and resistance genes to humans [43–46].

More than 93% of our survey students had chosen strong or moderate contribution about veterinary drug use to overall AMR what is in line with high awareness of AMR, among healthcare students across the Europe [47,48]. Contrary, the lower percentage of veterinary students in South Africa [49] and in Australia [39] (80% and 88%, respectively) believed that the misuse of antimicrobials by veterinarians contributed significantly to AMR. Our results showed that the main reasons for these contributions among surveyed students were irrational, excessive and irresponsible use of antimicrobials, especially in food animals, as well as the lack of C & S testing of samples. Moreover, most companion animal veterinarians placed greater importance on the contribution of AMU in livestock to AMR development [50,51] since awareness on this issue differs among veterinarians according to their type of practice [17,49].

In the last few years all countries have been encouraged to adopt national strategies, including Serbia [52] and Croatia [53] and to improve AMS programs in line with global strategies [25,54] where special attention is given on increasing awareness among those who prescribe antimicrobials. Our findings suggest that about half of students have heard about national strategies in both countries, giving promising results that they will rely on them in their professional work.

According to veterinarians' opinion, improvement of the biosecurity measures and strict control of specific infectious diseases are promising measures in AMU reduction [55]. As many as 70% of students of our study sample have heard about biosecurity guidelines in both countries, what could result in more proactive approach in communicating this issue to farmers and animal owners in their practice [56,57]. Furthermore, appropriate use of PPE procedure and biosecurity measure were reported for two thirds of our students in case of only for two scenarios, a cow with acute watery diarrhoea and a horse with same diagnosis. This suggests that giving more lessons on biosecurity at the undergraduate level could be beneficial in students' future work in order to combat AMR. In addition, the surveyed students who often referred to National Strategy showed a higher knowledge score on the level of biosecurity measures they would take in opting for and performing procedures in the range of clinical scenarios. These findings indicated that knowledge among our students on target actions to prevent AMU when animals are susceptible to disease should be raised to a higher level.

Prudent use of antimicrobials is corroborated by the WHO list of critically important antimicrobials for human medicine and rating of their importance due to reduction of AMU in food-producing animals [40]. More than 70% of our surveyed students rated amoxicillin correctly, while only 8.5% of them identified gentamicin correctly, both as first-line therapy. In Serbia and Croatia only amoxicillin, but not gentamicin, is licensed for the animal use. This suggests that our students were more familiar with amoxicillin, especially after clinical teaching. Chloramphenicol (second line) was incorrectly categorized by more than 70% of our students. Research results are similar with the study conducted in Australia, where the adverse effect of this drug on people and low use of this drug in clinical veterinary practice could have influenced that 63% of veterinary students believed that chloramphenicol had a higher importance rating [39]. In Serbia and Croatia use of chloramphenicol is prohibited in food-producing animals, which suggests that this could be a contribution to surveyed students’ opinion. Surveyed students who expected to graduate later were more likely to identify the importance of rating antimicrobials correctly than those who thought they would graduate earlier. This could mean that clinical teaching in the final year of their study did not influence the awareness regarding rating of antimicrobial importance. Contrary, veterinary students in Australia who expected to graduate in 2018 were less likely to identify this rating correctly than 2017 graduates [39].

Around 80% of our students said they would rarely or never use systemic antimicrobials in clinical scenario regarding 2-days old wound over the cannon in a horse, while even 97% of veterinary students in Australia would always or frequently use antimicrobials in this clinical scenario although there was no recommendation for use of antimicrobials in clinical guidelines for this indication [39,58,59]. These guidelines in veterinary medicine are scarce in Serbia and Croatia, but they are well established in Australia what suggests that this could be possible reason why students in Australia are more familiar with use of antimicrobials in different clinical scenarios.

Education, scientific literature and continued education are sources which influence prescribing behavior of veterinarians regarding AMU [60–62]. Our study revealed that those students who denied having good knowledge of the pharmacology of antimicrobials showed higher knowledge about systemic use of antimicrobials in different clinical scenarios. This disproportion in our students' knowledge has suggested that they need more clinical experience.

Zhuo et al. [16] showed that veterinarians were more likely than medical doctors to perceive lack of rapid diagnostic tests as a significant barrier in prescribing decisions. Cost of diagnostic testing, particularly C & S testing, led to overuse of antimicrobial in veterinary practice [17,50]. Nevertheless, veterinarians perceived that better public access to rapid diagnostic tests would be helpful in supporting more appropriate prescribing [16].

As for students’ opinion on C & S testing for a range of clinical scenarios, more than half of them choose it only in case of one clinical scenario (a dog with recurrent pyoderma). It is in accordance with the published guidelines for antimicrobial therapy due to the increased frequency of isolation of antibiotic resistant staphylococci in veterinary medicine [63]. Interestingly, a small number of surveyed students (9.4%) were aware that obtaining culture samples should be the mainstay of directed therapy wherever possible in scenario regarding a horse with distal limb cellulitis in a single leg, although *Corynebacterium equi* [64] and *Rhodococcus equi* [65] can be causes of cellulitis, and samples for C & S testing should always be submitted if possible [66]. Contrary, more than 80% of veterinary students in the United Kingdom [67] and 97% in South Africa [68] are aware that C & S testing is part of AMS principles. Our research results suggest that education on this subject has to be improved because a deficiency in veterinary curricula or lack of integration of knowledge. Our students who think they will graduate sooner have higher knowledge level on C & S testing sample submission for range of clinical scenarios; and these are the ones who have passed more preclinical and clinical subjects. It could be a reason why their knowledge on this issue is more adequate.

Insight in current antimicrobial prescribing behavior among veterinarians in companion [17, 69–71] and in production animals practice [72,73] is essential to perform before AMS implementation. The insight into the barriers in AMS by defining the issues that influence the prescribing behavior of veterinarians is provided in Australia [49]. According to our findings more than 95% of students reported having good understanding of AMS. In Australia, one survey [39] has shown that more than 80% of veterinary students, while another study [74] has shown that more than 60% of them perceived that statement. Contrary, in Nigeria a very low percentage of veterinary students (13.1%) [75] and veterinarians (17.1%) [76] have heard about AMS, as well as only about 24% of pharmacy students in Saudi Arabia [77]. Having appropriate knowledge on AMS at the undergraduate level could insure AMS implementation in future work of veterinarians.

Our study revealed that less than 50% of respondents agreed that prudent AMU was taught enough. Nevertheless, only 2.43% of veterinarians from different types of practice from seven different countries indicated formulary/prescribing guidelines for the factors influencing their prescribing behavior [61], although the implementation of AMU guidelines is one activities in promotion of prudent AMU [78].

Only 50% of surveyed students stated that they had good knowledge on the pharmacology of antibiotics and that the amount of teaching time for pharmacology was about right. Guardabassi and Prescott reported that reduction of inappropriate AMU and enhanced understanding of stewardship were linked with good education, but that teaching of AMR and antimicrobial pharmacology was inadequate in most university curricula [10]. More education on pharmacology was reported among the majority of veterinary students from Australia [39] and Nigeria [76]. Contrary, in South Africa 76% of them believed their ungraduated training prepared them well when choosing an ideal antimicrobial drug for an individual patient [49]. Medical students across the Europe [46,47], China [79,80] and the United States of America [81] reported they would prefer more education on antibiotics and AMR. Understanding of the mechanisms of AMR is an essential output in veterinary education curricula to achieve this. More than 86% of surveyed students reported having good understanding of the mechanisms of AMR what is in line with veterinary students in Nigeria [82] and Australia [39].

## Limitations

We did not approach the final year of the study at University of Novi Sad in Serbia since their sixth year curriculum was adopted in 2013 and introduced in 2014. Before that, they had five-year curriculum and when this survey was conducted, the sixth year students were not enrolled in the studies yet. In general, in this survey females were more represented than males. Moreover, the gender distribution of 67.6% of females (Table 1) in this study was similar to the one of veterinary students in Australia [75]. In addition, the gender distribution of surveyed veterinary students was similar at the University of Zagreb and the University of Novi Sad. Finally, although the sample size may be relatively small and includes only two universities, the results can reflect the pattern of knowledge and comprehension of antimicrobial stewardship and biosecurity among veterinary students in southeastern part of Europe.

## Conclusion

This study highlights that although students enrolled in veterinary medicine course in Serbia and in Croatia reported having good understanding of AMS, they have shown insufficient level of knowledge on AMU in clinical practice, as well as biosecurity measures and PPE procedure. Therefore, the first step in implementing AMS principles among veterinarians is to improve awareness of prudent AMU and AMU guidelines at the undergraduate level. The primary focus should be on delivering adequate educational interventions tailored with gaps, which were recorded in surveys such as this one. Hence, further research is needed to validate our findings on a larger sample which should include veterinary students from all European countries.

## Supporting information

S1 Questionnaire Questionnaire on antimicrobial stewardship survey. Copy of the questionnaire used in the study in English language.
(DOCX)
