# Supplementary material for: Mapping knowledge and comprehension of antimicrobial stewardship and biosecurity among veterinary students
Source: PLoS One. 2020 Aug 19;15(8):e0235866. doi: 10.1371/journal.pone.0235866 (PMC7446898; doi:10.1371/journal.pone.0235866)
Supplement: S1 Questionnaire — Copy of the questionnaire used in the study in English language. (DOCX) [file pone.0235866.s001.docx]

**Antimicrobial stewardship survey**


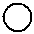
 Please complete the following survey.


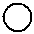
 Which university do you attend? University of Zagreb, Croatia

University of Novi Sad, Serbia

1. All going well, in which year will you graduate?

2020.


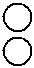


2021.


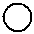
 2022.

1. What is your main area of interest?


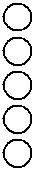


Companion animals

Equine

Bovine

A mix of the above

Public health, government, industry, research


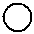
 Haven't decided yet

1. Do you think that veterinary use of antimicrobials contributes to overall antimicrobial resistance?

Strong contribution


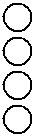


Moderate contribution

Minimal contribution

No contribution


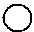
 Not sure

1. For the question above, what are the major reasons you answered this way?

__________________________________

1. **For each of the following antimicrobials, please indicate if they are for first, second or third line therapy:**

First line Second line Third line Not sure

1. Amoxicillin


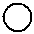

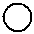

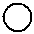

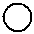


1. Amoxicillin clavulanate


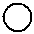

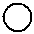

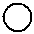

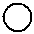


1. Amikacin


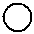

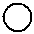

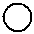

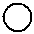


1. Oxytetracyclin


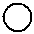

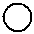

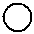

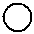


1. Metronidazole


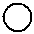

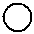

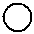

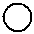


1. Enrofloxacin


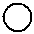

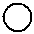

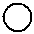

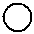


1. Procaine penicillin


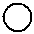

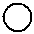

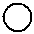

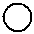


1. Cefovecin


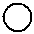

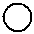

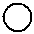

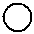


e. Trimethoprim sulphonamide


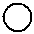

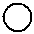

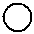

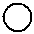


1. Gentamicin


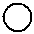

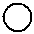

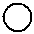

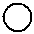


1. Vankomycin


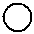

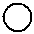

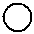

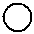


1. Clindamycin


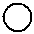

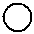

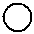

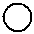


1. Chloramphenicol


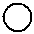

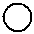

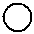

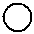


1. Cephalexin


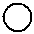

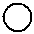

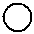

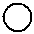


1. Marbofloxacin


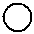

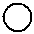

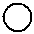

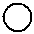


1. Rifampicin


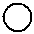

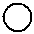

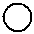

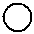


**6. For each of the following scenarios please indicate if you think systemic (injectable or oral) antimicrobials are indicated:**

Always Frequently Rarely Never Not sure

Routine dog spey


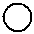

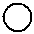

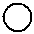

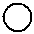

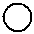


Pancreatitis in a dog


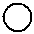

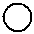

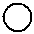

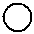

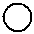


2 year old cat with stranguria


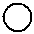

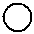

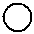

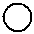

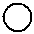


and haematuria

Lame cow


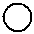

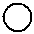

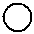

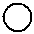

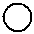


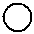
Routine gelding


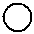

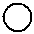

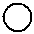

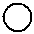


2 day old wound over the canon


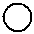

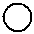

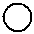
bone of a horse with bone

exposed

Cat with a draining abscess on

Its face

Routine dental prophylaxis in a dog

Upper respiratory tract disease

u of 4 days duration in an

otherwise healthy cat

Dog with superficial bacterial

dermatitis

3 week old calf with diarrhoea

Foal with patent urachus

Horse with fever of unknown origin

Dog with severe haemorrhagic

gastroenteritis but not sepsis

Cow that calved 2 days ago that

has retained foetal membranes

3 year old cow with moderate

mastitis in 1 quarter

Colibacillosis in a poultry flock

**7. For each of the following scenarios please indicate if you would submit samples for culture and susceptibility testing:**

Always

Frequently

Rarely

Never

Not sure

2 year old cat with stranguria

and haematuria

5 year old female dog with

stranguria and haematuria (first

occurrence)

5 year old female dog with

stranguria and haematuria that

has previously been treated with

amoxicillin clavulanate

4 week old foal with pneumonia

A group of 4 week old calves

with pneumonia

4 year old cow with mild mastitis

in 1 quarter

4 year old cow with gangrenous

mastitis (black mastitis)

*Otitis externa in a dog (1st*

occurrence)

*Recurrent otitis externa in a dog*

Adult cow with acute watery

diarrhoea

Horse with distal limb cellulitis in

a single leg

Calf with a septic hock joint

Foal with a septic hock joint

Pyothorax in a cat

Dog with recurrent pyoderma

Diarrhoea in grower pigs

Pneumonia in a group of feedlot

cattle

What are the 3 most important factors that would influence your decision to submit samples for culture and sensitivity?

Location of the infection

Ease of obtaining a samp

Persistent infection

Severe infection

Client finances

Unusual infection

Atypical cytology

To confirm a diagnosis

Notifiable diseases

Herd problems

Re-occurring infections

Other

Please specify:

__________________________________

**8. For each of the following scenarios please indicate the level of biosecurity you would take for examination and performing procedures:**

| None | Hand | Gloves | Gown/ove | Gloves & | Gloves, | Gloves, | Not |
| --- | --- | --- | --- | --- | --- | --- | --- |
|  | wash | Only | ralls only | gown/ove | gown/ove | overalls | sure |
|  | after |  |  | ralls | ralls & | with head |  |
|  | contact |  |  |  | protection | protection |  |
|  |  |  |  |  | (respiratory mask and goggles) | respiratory mask and goggles |  |
|  |  |  |  |  |  |  |  |
|  |  |  |  |  |  |  |  |
|  |  |  |  |  |  |  |  |
|  |  |  |  |  |  |  |  |

Routine examination of a dog or cat

Routine examination of a horse

Routine examination of a cow

Horse with fever of unknown

origin and neurological signs

Horse with acute watery

diarrhoea

Cow with acute watery diarrhoea

Cat flu of 4 days duration in an

otherwise healthy cat

*Post mortem* examination of a cow

*Post mortem* examination of a horse

Galah with respiratory disease

Aborted foetal material from a horse

Routine dental prophylaxis in a dog

Cow with dystocia (calving)

Sick bat

Goats with poor conception rates

Mare with dystocia (foaling)

Methicillin resistant

*Staphylococcus pseudintermedius*

*dermatitis* in a dog

An animal with a multi-drug

resistant urinary tract infection

When entering a pig farm

**9. Please indicate your level of knowledge about the following guidelines:**

Refer to often

Never heard of

Know of

Have read

Regulation on the National Program

to control bacterial resistance

for antibiotics in the Republic of Serbia

(or Republic of Croatia)

Regulation on recipe form and content

for veterinary drugs as well

publishing and prescribing

veterinary drugs method

Regulation about establishing

program of animal health measures

for 2019. year

**10. Please indicate how strongly you agree to each of the following statements:**

Strongly agree

Agree

Neither agree nor

Disagree

Strongly disagree

a. I understand what antimicrobial

stewardship is.

b. The amount of teaching time for

prudent antibiotic use is about right.

c. I understand antimicrobial

resistance mechanisms.

d. I have a good knowledge of the

pharmacology of antibiotics.

e. The amount of teaching time for

pharmacology is about right.

f. I know how to use antibiotics to

minimise the risk of antimicrobial resistance

developing.

g. What I have learnt about

antibiotic use in clinics is the

same as what is taught to me in

lectures.

h. What I have learnt in clinics is

more useful than what I learnt in

lectures.

Anything else you'd like to tell us?

__________________________________________

Thank you for completing the survey!
